# Morphologically, immunohistochemically and PCR proven lymphocytic viral peri-, endo-, myocarditis in patients with fatal COVID-19

**DOI:** 10.1186/s13000-022-01207-6

**Published:** 2022-02-17

**Authors:** Evgeniya Kogan, Yuriy Berezovskiy, Olga Blagova, Anna Kukleva, Lyudmila Semyonova, Evgeniy Gretsov, Atadzhan Ergeshov

**Affiliations:** 1 I.M.Sechenov First Moscow State Medical University (Sechenov University), 8-2, Trubetskaya street, Moscow, 119992 Russia; 2 Central Tuberculosis Research Institute, 2, Yauzskaya Alleya, Moscow, 107564 Russia

**Keywords:** COVID-19, Coronavirus, Morphological and immunohistochemical study, PCR, Myocarditis, Endocarditis, Pericarditis, Toll-like receptors

## Abstract

**Background:**

Despite a reported cardiac injury in patients with new coronavirus infection, the possibility and specifics of genuine viral myocarditis in COVID-19 remains not fully clear.

**Purpose:**

To study the presence of SARS-CoV-2 in the myocardium and the morphological properties of myocarditis in patients with severe coronavirus infection (COVID-19).

**Methods:**

Autopsy data of eight elderly patients (75.6 ± 7.4 years), four male and four female, with severe new coronavirus infection were studied. The lifetime diagnosis of COVID-19 is based on a positive result of the PCR study. The inclusion criterion was the presence of morphological signs of myocarditis according to the Dallas criteria. A standard histological examination included staining by hematoxylin and eosin, toluidin blue and Van Gieson. An immunohistochemical study was performed using antibodies to CD3, CD 68, CD20, perforin, toll-like receptor (TLR) types 4 and 9. PCR in real-time was performed to determine the viral RNA in the myocardium.

**Results:**

All patients had severe bilateral viral pneumonia. In all cases, myocarditis was not clinically diagnosed. Morphological examination of the heart found signs of active lymphocytic myocarditis. PCR identified the SARS-Cov2 RNA in all cases. There were also signs of destructive coronaritis in all cases, thrombovasculitis, lymphocytic pericarditis (in 3 cases) and endocarditis (in 2 cases). The absence of neutrophils confirms the aseptic nature of inflammation. An immunohistochemical study showed the CD3-positive T lymphocytes in the infiltrates. Increased expression of TLR type 4 and less 9 was also detected.

**Conclusion:**

Morphological and immunohistochemical evidence of myocarditis in COVID-19 was presented. Lymphocytic infiltrations and positive PCR confirm the viral nature of inflammation. Myocarditis in COVID-19 is also characterized by coronaritis with microvascular thrombosis and associated with lymphocytic endo- and pericarditis.

## Background

Heart damage is one of the typical manifestations of a new coronavirus disease (COVID-19). In a clinical study from Wuhan, myocardial damage was diagnosed in 12% of cases among 41 patients, [1]. There is a clear correlation between severe current and lethal outcomes with the cardiac symptoms, [2]. At the same time, the morphology of myocardial lesions in a new coronavirus infection is poorly known.

Different variants of heart lesion in COVID-19 are described. 1. Deterioration of chronic cardiovascular diseases, [3]. 2. Development of acute myocardial infarction due to thrombosis of both altered and intact coronary arteries, [4]. 3. Acute development of cardiogenic shock, severe heart failure in patients without previous heart disease, [5]. 4. Less severe symptoms (arrhythmias, ECG changes, other), [6]. The increase of biomarkers (troponin, NT-proBNP, etc.) is detected in 8% of patients, [2].

The term «acute myocardial injury» is most commonly used to explain cardiac symptoms and laboratory changes in COVID-19. According to the ESC Guidelines 2018, myocardial injury is defined when troponin is elevated without ischemia, [7]). His morphological evidence is the death of cardiomyocytes. This conception is too general, it seems. In the case of severe viral myocarditis, myocardial necrosis also would qualify for this definition. However, in case of coronavirus infection, clinicians try to avoid the term «myocarditis».

This happens due to the difficulties in the necessary morphological verification of myocarditis, [8]. At the time of this report, there are only two original morphologically verified cases of myocarditis in COVID-19, [9, 10]. At the same time, other mechanisms of myocardial affection are discussed: virus binding to angiotensin-converting enzyme-2 receptors; oxidative stress; lesion of the microcirculatory vessels (increased permeability, angiospasm, microtrombosis); systemic inflammatory response (cytokine storm), [3]. In addition, several drugs used in COVID-19 are cardiotoxic.

In this context, the possibility of true viral myocarditis, and also peri- and endocarditis in COVID-19 is still unclear. Therefore, this study is highly actual.

### Purpose

to observe lymphocytic myocarditis in patients with COVID-19 the lymphocytic myocarditis and study its morphological properties in patients with severe coronavirus infection (COVID-19).

## Methods

### Characteristics of patients

Autopsy data of eight elderly patients (from 63 to 84 years, 75.6 ± 7.4 years on average), four male and four female, with new coronavirus infection and bilateral polysegmental pneumonia were studied. The cause of death was progressive heart-pulmonary insufficiency and multi-organ failure.

The diagnosis of COVID-19 was established based on of typical clinical signs and a positive result of a nasopharyngeal smear study by PCR. All patients were treated in different hospitals reprofiled for COVID-19. The *reason for inclusion in the study* was the detection of myocarditis in autopsy according to international Dallas criteria, [8].

Previous clinical examination in COVID hospitals included repeated blood tests, chest computed tomography (CT), electrocardiography (ECG), echocardiography (EchoCG), investigation of troponin levels and inflammatory markers in the blood. COVID-19 treatment was performed according to protocols approved in each hospital. It included antimalarial (hydroxychloroquine), antiviral (lopinavir-rotanavir), antibacterial, anti-cytokine drugs (tocilizumab), anticoagulants and in some cases corticosteroids. All the patients in the final stage of the disease underwent invasive ventilation of the lungs.

### Morphological examination

A traditional morphological investigation was performed: autopsy with macroscopic description, histological examination of the heart with hematoxylin and eosin stains, toluidin blue and Van Gieson stains.

### Immunohistochemical (IHC) study

Serial paraffin slices were studied immunohistochemically by standard methods. A panel of antibodies to CD3, CD 68 (CellMarque, rabbit monoclonal antibodies, dilution titer 1:1000), CD20 (CellMarque, rabbit monoclonal antibodies, dilution titer 1:500), perforin (CellMarque, mouse monoclonal antibodies, dilution titer 1:50), toll-like receptor type 4 (TLR-4, GeneTex, rabbit polyclonal antibodies, dilution titer 1:200), TLR-9 (GeneTex, rabbit polyclonal antibodies, dilution titer 1:50) was used.

### Real-time polymerase chain reaction (PCR)

Wax block material was used. Total RNA was isolated from the obtained tissue fragments using RNeasy Mini Kit (Qiagen, Germany). Real-time PCR for SARS-CoV-2 detection was performed using the QuantiTect single-step PCR kit (Qiagen). The primers were selected based on publicly available DNA and mRNA sequences in the NCBI database using the Primer-BLAST program.

**Table:** 

Gene	5′-Primer	3′-Primer
*SARS-CoV-2*	WuhanCoV-spk2-f 5′-TTTCCTCGTGAAGGTGTCTTTGT-3 ‘	
	WuhanCoV -spk2-r 5′-TGTGGTTCATAAAAATTCCTTTGTG-3 ‘	
	WuhanCoV-spk2-hex-p5’-HEX-TCAAATGGCACACACTGGTTTGT-BHQ1	

## Results

### Some clinical findings of the patients

As previously noted, all patients were in the older age group. In addition to age, most patients had several risk factors for the unfavorable course of coronavirus pneumonia.

These include obesity (three patients), diabetes (two patients), arterial hypertension (six patients), coronary heart disease (two patients), various active oncological diseases (two patients) and surgical treatment of a tumor in the past (one patient), as well as long-term physical inactivity due to a femoral neck fracture in one patient. From the chronic diseases of the respiratory system, only the bronchitis of a smoker in one patient was indicated.

In all cases, the main clinical manifestations of coronavirus infection were febrile fever, cough and increasing shortness of breath. Patients were admitted to the hospital on days 6–10 after the onset of the fever. Death happened on days 10–14 from respiratory and multi-organ failure. In one case, cardiogenic shock was diagnosed as an immediate cause of death.

In a lifetime, the diagnosis of myocarditis was not suspected in any case. Therefore, no detailed cardiac examination was performed. In a patient with cardiogenic shock, the ECG showed ST segment elevation. In another case negative T waves, atrial flutter were observed. Two patients had elevated troponin T levels in the blood. Blood tests showed the typical for COVID-19 lymphopenia, a dramatic increase in the levels of inflammatory markers, D-dimer, lactate dehydrogenase, creatine kinase. Left ventricular hypertrophy, a moderate decrease in contractility, and also pulmonary hypertension were observed on EchoCG in some cases.

### Results of the macroscopic examination

There were no cases of over fluid in the pericardial and pleural cavities.

Macroscopic cardiac examination showed dilatation of the chambers, wall clots usually in the right atrium and ventricle, hypertrophy of the left ventricle wall. Cardiac weight ranged from 310 to 430 g. The wall thickness of the left ventricle was up to 1.4–1.9 cm. Wall thickness of the right ventricle was 0.3–0.5 cm. Myocardium had a flabby consistency. The section showed small yellowish reddish speckles. In some cases, fine hemorrhages were detected visually.

All patients showed signs of coronary atherosclerosis. However, only two patients had stenoses above 50%. Thromboses of the pulmonary arteries of different caliber were found in 100% of cases. One patient each also had ischemic brain and spleen infarcts.

### Results of virological study by PCR

Real-time PCR identified the SARS-Cov2 RNA in the myocardium in all cases. The present study did not include a comparison of data on virus persistence in the myocardium and other organs and tissues. However, such a study was performed in some patients, and RNA of the coronavirus was also detected in the lung tissue.

### Signs of myocarditis in histological examination

**Fig. 1:**
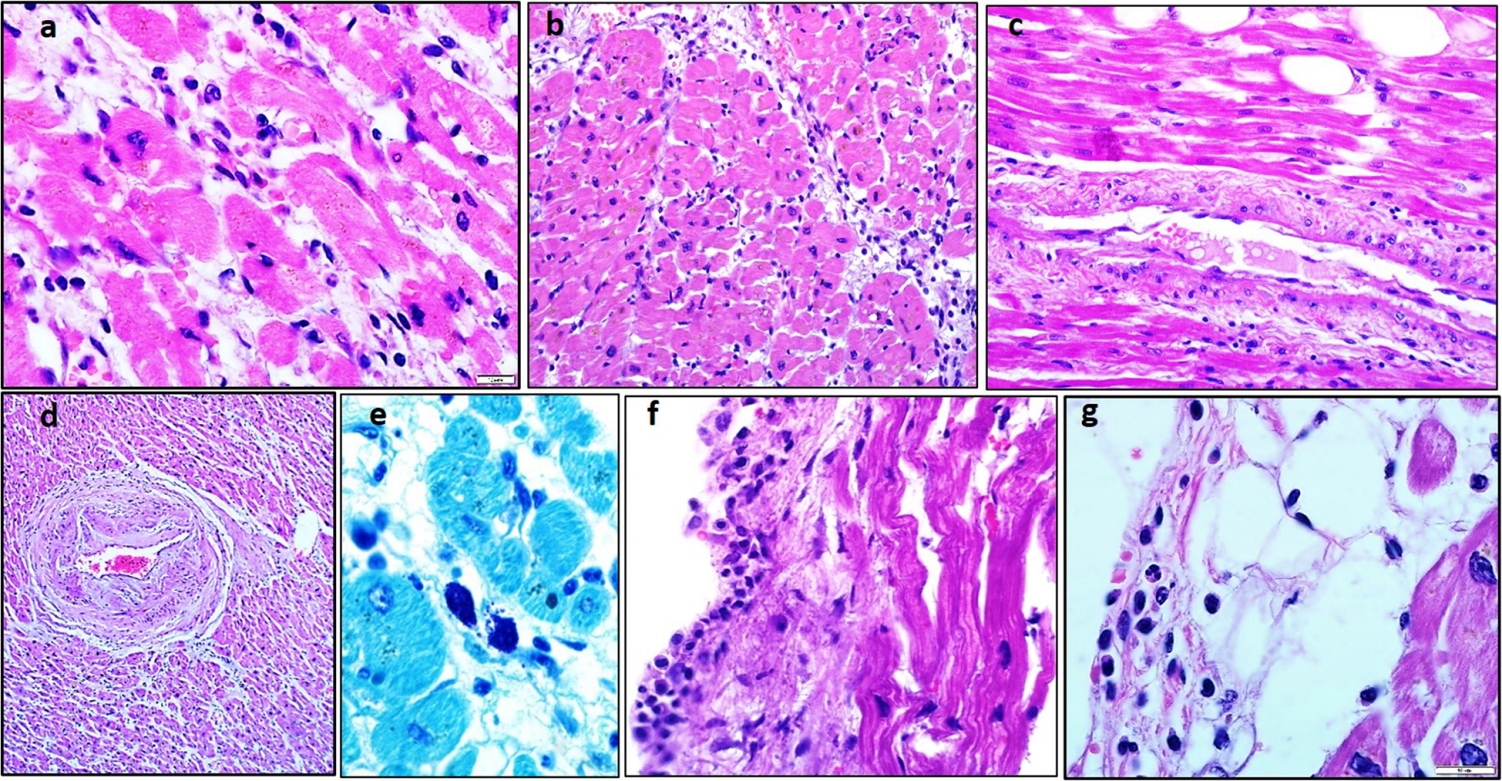
Histological characteristics of myocarditis in COVID-19. **a,b** - cardiomyocyte degeneration, diffuse lymphocytic infiltration in myocardium stroma; **c,d** - destructive productive coronaritis with intravascular thrombosis of small branches, **e** - degranulation of mast cells in myocardial stroma, **f** - lymphocytic endocarditis, **g** - lymphocytic pericarditis. Staining by hematoxylin and eosin (a-d,f,g) and toluidin blue (e), magnification × 600 (a,e,f,g), × 200 (b,c,d)

In the microscopic examination of myocardium interstitium is nonuniformly expanded, edematous with the lymphocytic-macrophage infiltrates (more than 14 lymphocytes, Fig. 1a and b) and lipomatosis. In toluidin blue, infiltration contains single mast cells with signs of degranulation (Fig. 1e). In one case, sporadic polymorphonuclear leukocytes were found in lymphocytic infiltrates. Cardiomyocytes are irregularly hypertrophied, with signs of overstrain, dystrophic changes, necrosis and lipofuscin deposits (Fig. 1a). Cardiomyocyte nuclei are saved, hyperchromic, with karyopyknosis. In some cardiomyocytes lysis and fragmentation of nuclei and cytoplasm are observed.

Small branches of coronary arteries in a state of destructive-productive vasculitis (coronaritis). Fresh blood clots were found in the gaps (Fig. 1c and d). Endothelial cells of large and small vessels are swollen, proliferating (expressed endothelitis). In all cases, hemorrhages were found. In one case, small areas of myocardial calcification were detected.

### Signs of endocarditis in histological examination

In two patients myocarditis was combined with lymphocytic endocarditis (Fig. 1f) and in three patients - with lymphocytic pericarditis (Fig. 1g). The absence of neutrophils in infiltrates suggested aseptic inflammation. One patient also showed flat parietal thrombotic masses, which correlated with intravascular thrombosis. The morphological picture, in this case, followed a non-bacterial thromboendocarditis.

### Signs of myocarditis in histological examination

In three patients myocarditis was combined with lymphocytic pericarditis (Fig. 1g). Neutrophils in the infiltrates were also absent. There were no data on in vivo detection of pericardial effusion or other clinical symptoms of pericarditis in these patients.

### Results of the cardiac immunohistochemical study of the myocardium

To confirm the diagnosis, an IHC study with a panel of antibodies to CD3, CD20, perforins, TLR-4, TLR-9 was also performed.

**Fig. 2:**
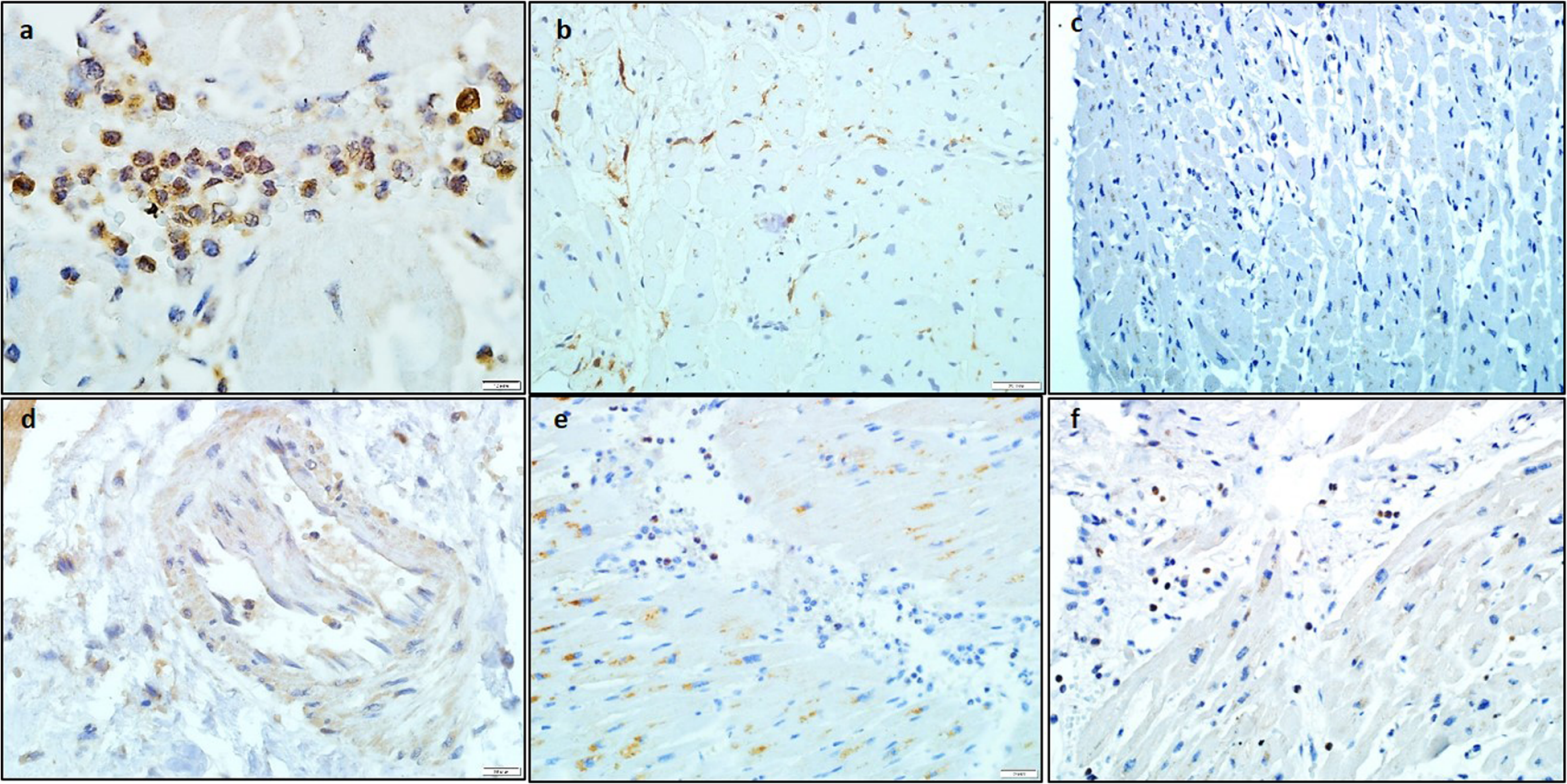
Immunohistochemical characteristics of myocarditis in COVID-19. **A** - CD 3 lymphocytes in myocardial stroma (more than 7 CD3+ lymphocytes per 1 mm^2^), **B** - CD68 macrophages in myocardial stroma, **C** - absence of CD20 expression, **D** - pronounced TLR 4 type expression in cardiomyocyte cytoplasm, endothelium and infiltration cells; **E** - weak TLR 9 type expression in cardiomyocyte cytoplasm, single macrophages; **F** - absence of NK cells (perforin expression). Immunoperoxidase method with diaminobenzidine, magnification × 600 (a,d), × 200 (b,c,e), × 100 (f)

A pronounced expression of CD3+ lymphocytes in interstitial space (more than 7 cells) and in thrombotic masses was detected (Fig. 2a). CD20+ B-lymphocytes were absent in all cases (Fig. 2c). A moderate number of CD68+ macrophages were also found (Fig. 2b). Perforin expression was low (found in only about 25% of infiltration cells, Fig. 2f).

There was observed a pronounced expression of type TLR4 in the cytoplasm of all cardiomyocytes, lymphocytic, macrophage and leukocytic infiltration elements, endothelial cells of vessels, pericytes, and smooth muscle cells of the vascular wall (Fig. 2d). A weak reaction of the cytoplasm of cardiomyocytes and certain leukocytes was also detected on TLR type 9 (Fig. 2e).

As a result, the diagnosis of active lymphocytic myocarditis was confirmed in all the patients included in the study.

## Discussion

This report demonstrates the number of morphologically, immunohistochemically and virologically proven cases of acute lymphocytic myocarditis (in combination with endo-, pericarditis) as part of a new coronavirus infection (COVID-19).

First of all, it is necessary to note the lymphocytic nature of myocarditis that we identified. This variant of infiltration is typical for viral myocarditis, but at the same time, it can indicate the role of immune reactions in the development of inflammation. The detection of coronavirus RNA in the myocardium of all patients supports the predominantly viral nature of myocarditis, which developed during the acute phase of severe coronavirus pneumonia. The increased expression of TLR types 4 and 9 in the myocardium, which we detected, also indicates the activation of innate antiviral immunity.

A specific feature of COVID-19 myocarditis is its combination with coronaritis. The injury of myocytes in this situation is rather complex and is based on several mechanisms: direct viral damage, ischemic damage due to coronaritis and coagulopathy and at last, it may be connected with cytokine storm. We can hypothesize a significant aggravation of cardiomyocyte ischemia in those patients in whom thrombi were detected in the cardiac microvessels. The morphological equivalent of ischemia can be considered severe dystrophic changes of cardiomyocytes, lysis and fragmentation of nuclei and cytoplasm. Polymorphonuclear leukocytes in infiltrates of one of the patients may reflect a response to ischemic cell death.

The frequency of myocarditis diagnosed clinically evaluated as 4.8%, [10]. The authors from Lombardy presented one of the first clinical cases classified as myopericarditis, [5]. A 53-year old woman without previous heart disease, a week later the respiratory infection developed systolic dysfunction (a decrease in the EF to 35%). MRI revealed diffuse myocardial edema and pericardial effusion, increased levels of highly sensitive troponin (0.59 ng/ml) and NT-proBNP (8465 pg/ml) were registered. Despite the absence of pneumonia, the diagnosis of COVID-19 was confirmed. Endomyocardial biopsy was not performed. Treatment with cardiotropic, antiviral drugs, steroids and chloroquine had a remarkable success. We also observed pericarditis in three patients with myocarditis. In one case, it was associated with atrial flutter, which is not unusual, [11].

Other cases of myocarditis diagnosed by MRI were described, including in young patients without pneumonia [12, 13]. This may indicate the specific genetic vulnerability of the myocardium to coronavirus and requires a special study. Fulminant myocarditis has been reported in several clinical cases. The authors from Wuhan used national criteria for myocarditis diagnosis, which do not require morphological verification, [14, 15]. Despite massive therapy with antiviral drugs, prednisolone, iv immunoglobulin, and the recovery of myocardial contractility, the patient died a month later from secondary infection. Unfortunately, the results of the autopsy are not reported. In a 59-year-old SARS-CoV-2-positive Spanish woman without pneumonia the diagnosis of myocarditis was based on acute heart failure, ST segment elevation, high troponin, myocardial edema, pericardial effusion, [16]. Biopsy was also not performed.

However, the most interesting are the results of morphological study in patients with suspected myocarditis. A more recent case is the death of a 17-year-old patient from cardiac arrest, followed by weakness, headache, nausea and vomiting in 2 days, [17]. The PCR study of the nasopharyngeal smear on COVID-19 was positive. No blood tests were performed while alive. An autopsy showed no signs of inflammation in the lungs, but eosinophilic myocarditis was diagnosed. Eosinophilia of bone marrow, signs of vasculitis, fibrinoid necrosis, thrombosis, granuloma, significant fibrosis were not found.

This tragic case is extremely unusual in consideration of the young age of the patient, the rapid development of symptoms, the absence of pneumonia and eosinophilic nature of myocarditis. It was also not established the fact of drug or narcotics abuse by the patient. It may be supposed a very special predisposition of this patient to such severe allergic reaction of the myocardium. The role of the coronavirus as a trigger for myocarditis is not clear here.

Another brief report presents a patient with a Tacotsubo syndrome, [9]. More than seven CD3-positive lymphocytes were found in the endomyocardial biopsy. But the virus itself was not detected in the myocardium. Experts from Germany have identified at least six scenarios of myocardial-like symptoms in COVID-19, including myocardial dysfunction due to cytokines and antibodies in the absence of the virus [11]. The cytokine storm may play a leading role in the Takotsubo syndrome. That doesn’t really indicate genuine myocarditis.

The results of endomyocardial biopsy in a 69-year-old COVID-positive patient were presented from Lombardy, [18]. A typical signs of severe myocarditis were associated with pneumonia. The coronary arteries were intact. The biopsy showed low-grade interstitial and endocardial inflammation only. The ultrastructural study revealed viral particles with coronavirus morphology in altered macrophages in the absence of them in the monocytes and endothelium. In cardiomyocytes only myofibrill focal lysis was observed. No signs of necrosis, vasculitis, severe fibrosis were identified. The patient died, but the autopsy details have not been reported.

This case does not meet the standard criteria for myocarditis. However it shows other possible mechanisms of cytopathic action of coronavirus on the heart. The authors discuss the possibility of the migration of alveolar macrophages to other tissues and organs. Nevertheless, the cases of isolated myocardial injury cited here make this hypothesis doubtful. The possibility of long-term persistence of the coronavirus or its trigger role with rapid elimination requires further studies.

An important aspect of the coronavirus myocarditis reported here is the detection of viral RNA in all patients. Until recent publications from the Charité clinic, there was no lifetime detection of coronavirus in the myocardium by PCR. There is only evidence of virus presence in the myocardium from autopsies [19]. The viral genome was detected in lung tissue in 100% of cases, in blood in six from 12 patients (< 4 × 10^4^ copies/mL) and in tissues (including the heart) in five patients with viraemia. In another autopsy study with PCR, the virus was found only in the lungs and was absent in other affected organs, [20]. In a study of 39 autopsy cases, the genome SARS-Cov2 was detected in the myocardium in 61.5% of cases, [21]. In 41% of cases, the viral load exceeded 1000 copies per μg RNA. There was no connection between the viral load and the presence of inflammatory infiltrates in the myocardium, which allows thinking about special conditions for the development of an inflammatory response to the virus.

Finally, in the study from Berlin among 104 patients with unknown cardiomyopathy, a genome of SARS-Cov-2 was revealed in the myocardium in five patients, [22]. These patients were diagnosed with lymphocytic myocarditis or inflammatory cardiomyopathy, whose features are similar to those described by us. However, these cases differ from ours in the longer course of cardiac disease. In our patients, the course of myocarditis remained unknown due to their early death from multi-organ failure. The lifetime studies indicate a low level of viraemia (about 10%), [23]. There is evidence that viraemia is common in patients with a more severe course of the disease and extrapulmonary lesions, [24]. It seems quite logical. Nonetheless we strongly believe that the absence of the coronavirus genome in the myocardium should not compromise the direct link between myocarditis and COVID-19. In these cases, one can suggest a transient action of the virus on the myocardium with initiation of long-term autoimmune reactions. It is quite typical for viral-immune myocarditis.

Thrombosis of both large and small coronary vessels seems to be one of the key mechanisms of myocardial injury. Non-inflammatory changes in the myocardium in patients with COVID-19 also included a weak lymphocytic infiltration in patients with respiratory distress syndrome [25], infiltration with a small number of monocytes and CD34-positive cells and interstitial fibrosis, [20]. These signs are considered markers of chronic myocardial injury.

Endo-, pericarditis in of COVID-19 with lymphocytic infiltration in our patients may have also viral and immune etiology. Whereas cases of exudative pericarditis are not difficult to diagnose clinically, the non-bacterial endocarditis we found is very difficult to suspect. Its latent course is supported by extensive use of anticoagulants in COVID-19, which can prevent thromboembolic complications.

However, in our cases, the changes were more significant than described before. They fully met the morphological and immunohistochemical criteria of true acute lymphocytic myocarditis. High expression of TLR4 in the lymphocytes, endothelium and cardiomyocytes indicates the involvement of congenital immunity and, especially, cytokine storm with myocardium injury. Less pronounced expression of TLR 9 may be related to their preferential activation by DNA-containing infective agents (SARS-CoV-2 is RNA-virus). Activation of the innate immune system may explain the maintenance of inflammation even after the virus elimination from the myocardium.

### The limitations of the study

The post mortem nature of the myocardial examination did not allow us to compare the morphological data with the clinical picture in detail. In particular, we were not able to correlate cytokine levels with changes in myocardium for all patients. In addition, we selected only patients with signs of myocarditis and did not analyze other forms of myocardial damage in virus-positive patients.

## Data Availability

Not available.

## Abbreviations

COVID-19: coronavirus infectious disease 2019
ECG: electrocardiogram
NT-proBNP: N-terminal prohormone of brain natriuretic peptide
PCR: polymerase chain reaction
SARS-CoV-2: severe acute respiratory syndrome coronavirus 2
TLR: toll-like receptor
